# Supplementary figures and images for: Conditional knockdown of transformer in sheep blow fly suggests a role in repression of dosage compensation and potential for population suppression
Source: PLoS Genet. 2021 Oct 18;17(10):e1009792. doi: 10.1371/journal.pgen.1009792 (PMC8553175; doi:10.1371/journal.pgen.1009792)

# Tetracycline-repressible female transformation system

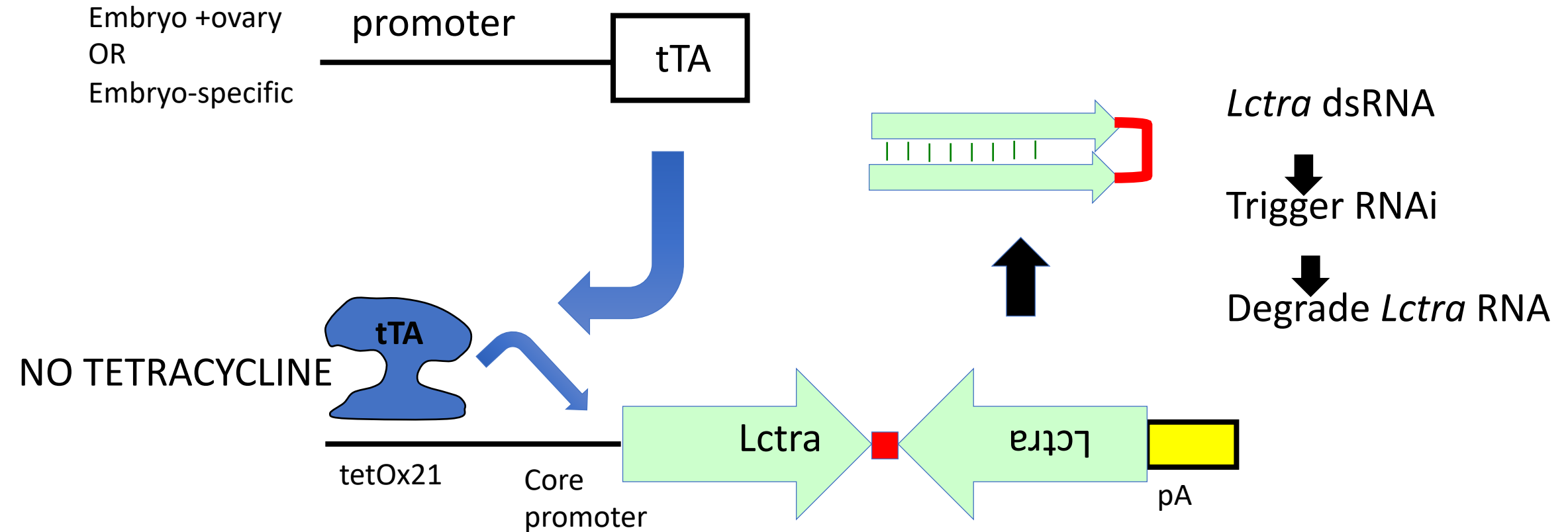

Supplement: S1 Fig — When strains that are homozygous for both a tTA driver and Lctra RNAi effector are raised on diet without tetracycline, tTA will bind to tetO and induce transcription of the Lctra inverted repeat gene. The hairpin RNA that is produced will self-anneal to form a long double-stranded RNA (dsRNA). The presence of the dsRNA will trigger an RNAi response and subsequent degradation of the Lctra mRNA. Without sufficient LcTRA protein, XX individuals will develop as males. The system is repressed by the addition of tetracycline to the diet, which inhibits the binding of tTA to tetO. One of the tTA drivers, DR3, has significant tTA expression in ovaries and embryos. Consequently, for production of transformed XX males, tetracycline was omitted from both the maternal and larval diets. (PDF) [file pgen.1009792.s001.pdf]

Wild type Pupae

DR3#6 LctraIR7 Pupae

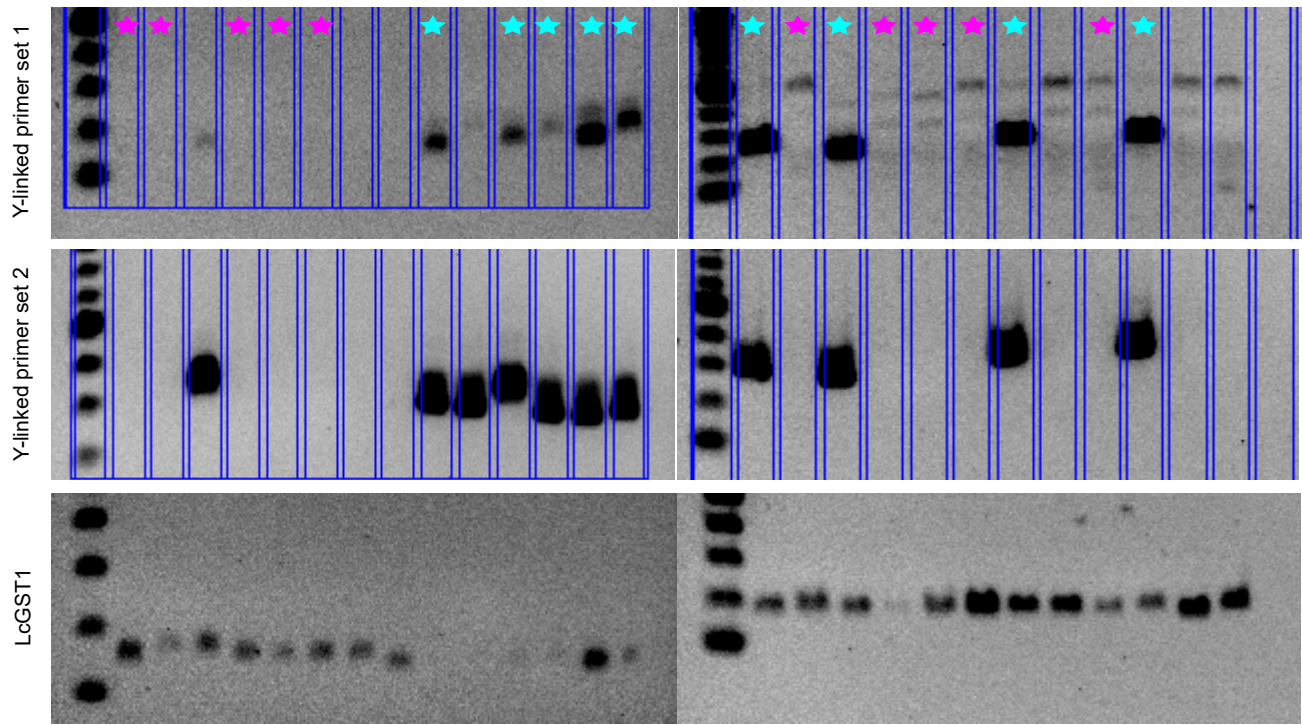

Supplement: S2 Fig — Genotyping of DNA from single mid-staged pupae DNA/RNA preps using two Y-linked primer sets. Lanes with a pink star indicate the five XX samples for both DR3#6; LctraIR7 and wild type used for RNAseq and qRT-PCR analysis. Lanes with a blue star indicate the five XY samples for both strains used in RNAseq and qRT-PCR analysis. DR3#6 LctraIR7, sample five, is missing from this genotyping as it was collected and processed on a different day. LcGST1 was used as a control primer set, specifically as a control for determining XX flies, as the XX flies are determined by lack of a band in the Y-linked primer set PCRs. (PDF) [file pgen.1009792.s002.pdf]

A

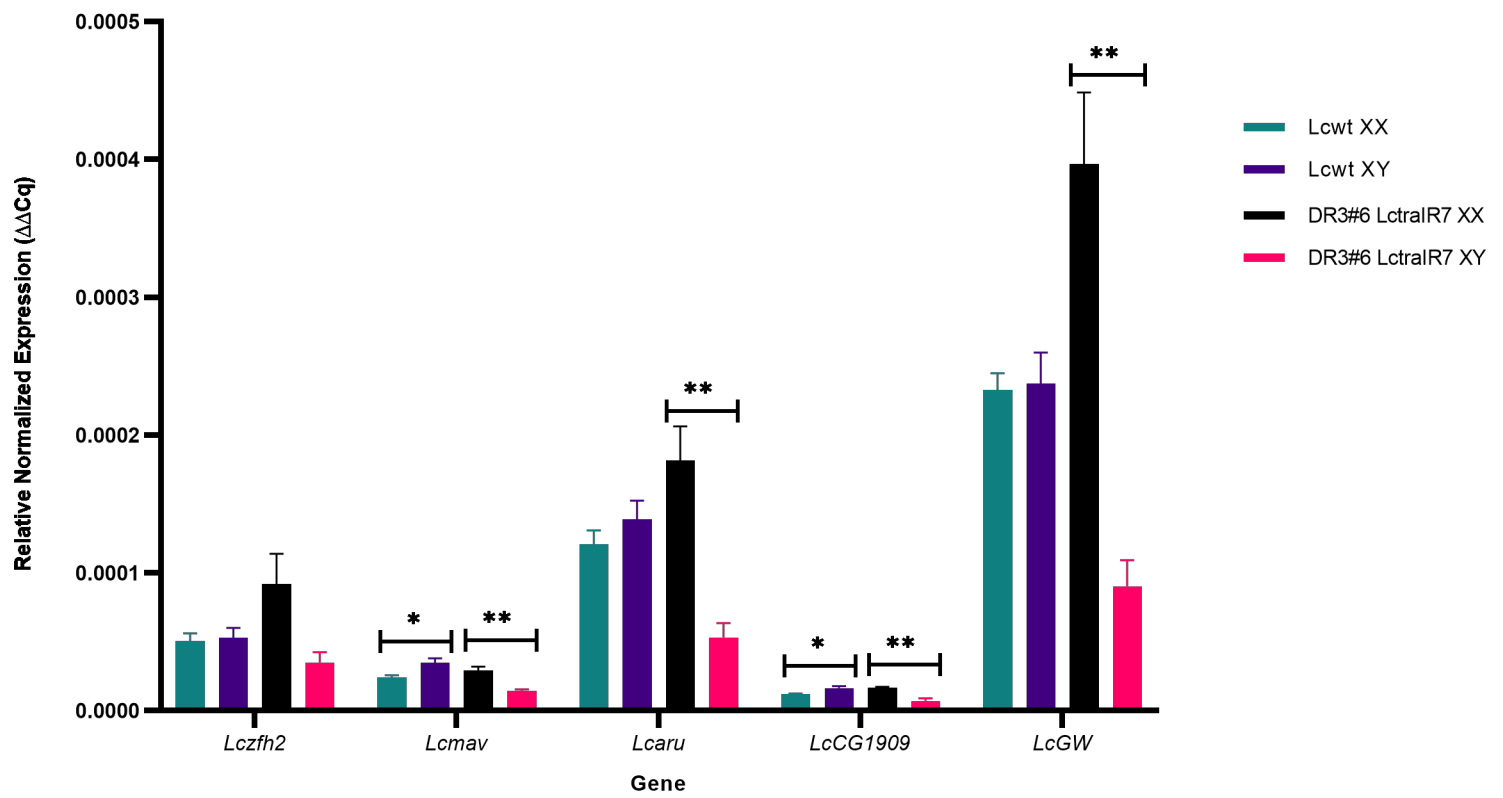

B

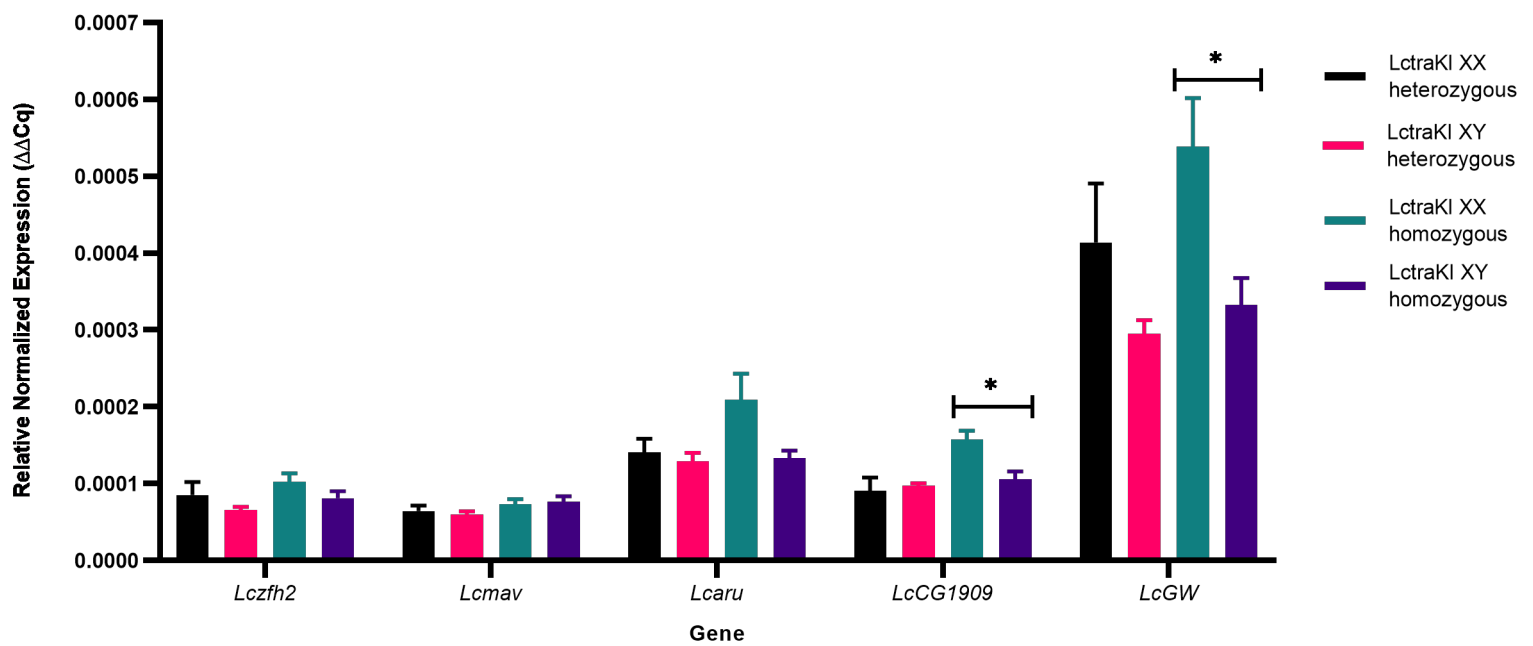

Supplement: S3 Fig — (A) qRT-PCR analysis of RNA levels for five X-linked genes in DR3#6; LctraIR7 or wild type XX and XY mid-staged pupae. Expression of X- linked gene is higher in DR3#6; LctraIR7 XX than XY pupae for all genes except Lczfh2 (Lcaru, P = 0.008; LcCG1909, P = 0.007; LcGW, P = 0.005; LcMav, P = 0.007; LcZfh2, P = 0.071; Student’s t-test). Expression of X-linked genes is higher in wild type XY pupae than XX pupae for two of the five genes (Lcmav, P = 0.025; LcCG1909, P = 0.039; Student’s t-test) Transcript levels were normalized to 28S rRNA. Expression levels were averaged over three biological replicates (DR3#6 LctraIR7) or 4 biological replicates (wild type) and standard error shown. Significance denoted on graph as * P < 0.05 or ** P < 0.01. (B) qRT-PCR analysis of RNA levels for five X-linked genes in LctraKI heterozygous or homozygous XX and XY hemi-sected adults. Expression of X-linked genes in LctraKI homozygous XX compared to XY is higher in two out of the five genes tested (Lcaru, P = 0.074; LcCG1909, P = 0.015; LcGW, P = 0.029; LcMav, P = 0.701; LcZfh2, P = 0.536; Student’s t-test). No significance was observed comparing LctraKI heterozygous XX and XY adults, however LcGW expression was visibly increased in LctraKI heterozygous XX flies (P > 0.05; LcGW, P = 0.183; Student’s t-test). Transcript levels were normalized to 28S rRNA. Expression levels were averaged over four biological replicates and standard error shown. Significance denoted on graph as * P < 0.05. (PDF) [file pgen.1009792.s003.pdf]

A

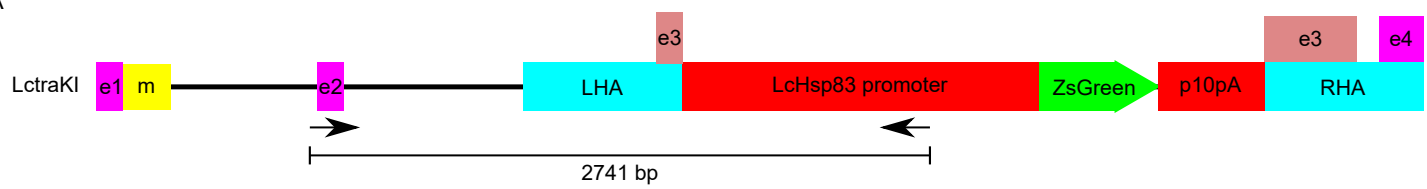

B

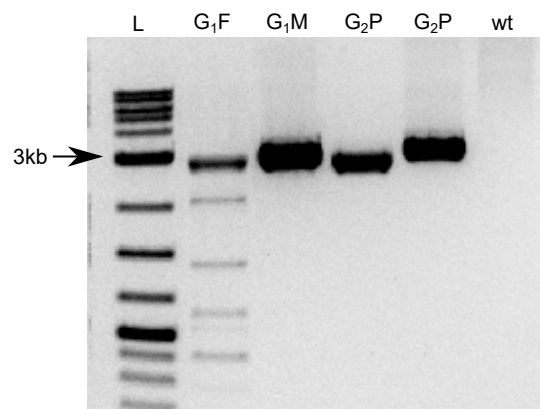

Supplement: S4 Fig — (A) Schematic of LctraKI into the genome. Knock in location disrupted exon 3 splitting it into two sections. Primers were designed within Lctra exon 2 and LcHsp83 promoter. When KI is integrated into the proper location in the genome, a band of 2714 bp will be produced. If KI is integrated into an off-target area, no band will be produced. (B) PCR analysis of genomic DNA from G1 adult female, G1 adult male, two G2 pupae, and wildtype control. The G2 pupae were generated from the G1 male crossed with wild type females and the LctraKI line was generated from this cross. All G1 and G2 samples amplified the proper band (2714 bp), while the wildtype control was negative as expected. Additional non-specific banding was seen in the G1 adult female. (PDF) [file pgen.1009792.s004.pdf]

A

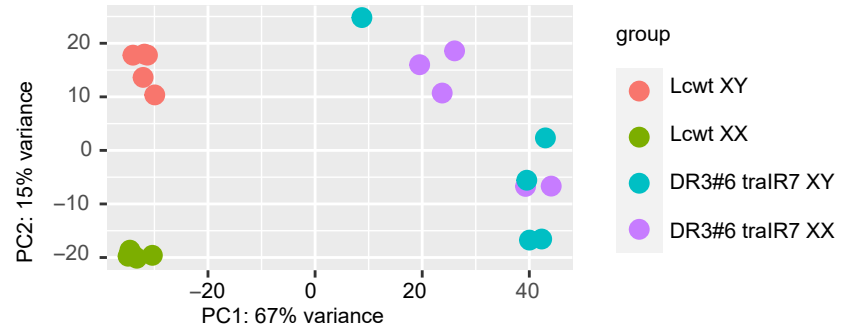

B

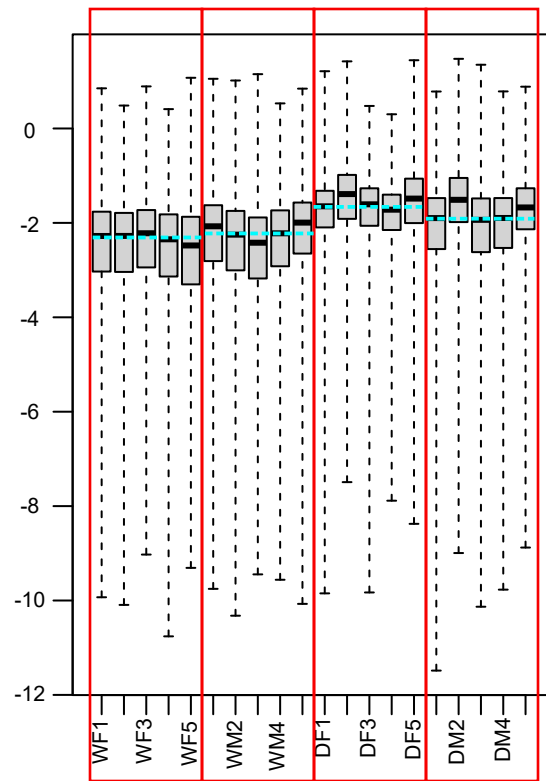

Supplement: S5 Fig — (A) Principal components analysis (PCA) of all 15 samples submitted for RNA sequencing. Both XX and XY wildtype samples grouped together as expected. The DR3#6 LctraIR7 samples were not as tightly grouped, with one DR3#6 LctraIR7 XY sample appearing out of place and two DR3#6 LctraIR7 XX samples grouping together but far from the remaining three samples. (B) Cook’s Distance Plots of each sample submitted for RNA sequencing. Samples were grouped by sample type (wild type XX, WF1-5; wild type XY, WM1-5; DR3#6 LctraIR7 XX, DF1-5; DR3#6 LctraIR7 XY, DM1-5) and lines drawn through the center of each sample type to help detect possible outliers. DF2, DF5, and DM2, together with the information from the PCA plot are considered outliers in our dataset and were not further used. (PDF) [file pgen.1009792.s005.pdf]
