# Supplementary material for: Conditional knockdown of transformer in sheep blow fly suggests a role in repression of dosage compensation and potential for population suppression
Source: PLoS Genet. 2021 Oct 18;17(10):e1009792. doi: 10.1371/journal.pgen.1009792 (PMC8553175; doi:10.1371/journal.pgen.1009792)
Supplement: S1 Table — (DOCX) [file pgen.1009792.s006.docx]

S1 Table Transformation efficiencies for generating LctraIR and LctraKI lines

| **Construct** | **Injection mix** | **# G_0_** | **# G_0_ founders** | **# Lines generated** | **Transformation efficiency** | **KI efficiency** |
| --- | --- | --- | --- | --- | --- | --- |
| pBac-tetO-LctraIR | pBac DNA + RNA helper | 93 | 5 | 5 | 5.3% | NA |
| pBS-traKI | Cas9 protein + tra sgRNA | 20^a^ | 2 | 1 | NA | 10% |

^a^ Only male G_0_ flies were selected for backcross to wildtype due to mutation in female genitalia
