# Supplementary material for: Conditional knockdown of transformer in sheep blow fly suggests a role in repression of dosage compensation and potential for population suppression
Source: PLoS Genet. 2021 Oct 18;17(10):e1009792. doi: 10.1371/journal.pgen.1009792 (PMC8553175; doi:10.1371/journal.pgen.1009792)
Supplement: S2 Table — (DOCX) [file pgen.1009792.s007.docx]

**S2 Table Primer Sets Used**

| **Oligo** | **Sequence 5’ 🡪 3’** | **Purpose** |
| --- | --- | --- |
| LctraIR1F_XN | AAATCTCGAGCCATGGTTCGACAGAATCAAGTTCACCGGAAAGATATCGTC | Generate Fragment 1 of LctraIR with XhoI and NcoI added to 5’ end |
| LctraIR1R_A | AAATCCTAGGCTGAGGTGTTGCGGGTATAATAGCAGTTGTTAAGG | Generate Fragment 1 of LctraIR with AvrII added to 5’ end |
| LctraIR1F_H | GTTGAAGCTTCGACAGAATCAAGTTCACCGGAAAGATATCGTC | Generate Fragment 2 of LctraIR with HindIII added to 5’ end |
| LctraIR1R_N | GTTGGCTAGCTGAGGTGTTGCGGGTATAATAGCAGTTGTTAAGG | Generate Fragment 2 of LctraIR with NheI added to 5’ end |
| Lctra_sgRNA_fwd | GAAATTAATACGACTCACTATAGGTAAGACACCGGAAAGATCTGTTTTAGAGCTAGAAATAGC | *tra* specific primer for gRNA template preparation |
| LHA_fwd | TGGAGCTCCACCGCGGTGGCCCGAAGTGCTTCAAATTC | Clone left homology arm |
| LHA_rev | AGATCCTTCGCTTTCCGGTGTCTTACGTC | Clone left homology arm |
| hsp83_ZsG_fwd | CACCGGAAAGCGAAGGATCTGGTTCATAACATAG | Clone ZsG marker for KI |
| hsp83_ZsG_rev | TCTAGAAGCTTCAGGGCAATGCAGATCC | Clone ZsG marker for KI |
| p10pA_fwd | ATTGCCCTGAAGCTTCTAGAATGAATCG | Clone p10pA for KI |
| p10pA_rev | CTGCGCCCAGGTTAACTCGAATCGCTATC | Clone p10pA for KI |
| RHA_fwd | TCGAGTTAACCTGGGCGCAGTGAAAGAC | Clone right homology arm |
| RHA_rev | CGAGGTCGACGGTATCGATACCGAGTTTGATAGTTGTTGTTGC | Clone right homology arm |
| tra_e2_H83_fwd | CGCAATACGGGCTGTAAAAC | Confirm location of insert in genome |
| tra_e2_H83_rev | GTTAGCAGCCATGCACTGAA | Confirm location of insert in genome |
| tra_qPCR_fwd | TCAAACAACCCTAGATGCCCG | qRT-PCR of tra gene |
| tra_qPCR_rev | ATTAGTTTTACAGCCCGTATTGCGC | qRT-PCR of tra gene |
| Y_spec_set1_fwd | TTCTGAGGGGACGTATGGTT | Genotyping primers- Y-linked set 1 |
| Y_spec_set1_rev | AGGTCCATAATTGATCCTACCCC | Genotyping primers- Y-linked set 1 |
| Y_spec_set2_fwd | TTTCACACTCATCGGACGGA | Genotyping primers- Y-linked set 2 |
| Y_spec_set2_rev | GGTCAGGGCCAAATGTGTTT | Genotyping primers- Y-linked set 2 |
