# Supplementary material for: Conditional knockdown of transformer in sheep blow fly suggests a role in repression of dosage compensation and potential for population suppression
Source: PLoS Genet. 2021 Oct 18;17(10):e1009792. doi: 10.1371/journal.pgen.1009792 (PMC8553175; doi:10.1371/journal.pgen.1009792)
Supplement: Attachment — Submitted filename: Response to reviewers.docx [file pgen.1009792.s010.docx]

Referee comments PLOS Genetics (PGENETICS-D-21-00775)

Reviewer's Responses to Questions

*Our responses are in italics*

**Comments to the Authors:
Please note here if the review is uploaded as an attachment.**

Reviewer #1: This paper describes the development of transgenic strains of Lucilia cuprina, a major pest of sheep with significant economic and animal welfare impact in Australia. By knocking down Lctra, a central gene in the sex determination pathway (and for which homologues are known in a range of other insects, following original discovery and characterisation in Drosophila), the authors show that tra is necessary for dosage compensation, which is the process by which Drosophila, mosquitoes, and some other XY/XX organisms cope with having half as many copies of the X chromosome/cell in males as in females. Lctra knockdown, or mutation by knock-in, leads to various degrees of female-to-male transformation, but in stronger cases also male-type dosage compensation and death.
As the authors note in at least one regard, little of this is surprising since it generally corresponds to results from other organisms. But expecting something and seeing it are not the same thing, and this work is competently done in an economically important pest insect. Nor should one underestimate the work involved in such studies on non-model insects.

Though I am not particularly expert in this regard, I think the most novel aspect relates to the effect on dosage compensation. This pathway separates from phenotypic differentiation surprisingly (to me) high up the pathway and it is not necessarily easy to predict which regulatory genes will or will not influence it. I think that in Medfly, similar XX pseudo-males, sex-reversed by knockdown of the Medlfy tra homologue Cctra, are viable and fertile. If that is indeed the case, the authors might make more of this difference [papers from Giuseppe Saccone].

*It is a little difficult to compare as we don’t yet know if X-linked genes are dosage compensated in medfly and if so, if disruption of dosage compensation is essential for males. Nevertheless, Giuseppe Saccone and colleagues can routinely produce viable XX males by injection of Cctra dsRNA. We have not been as successful with Lctra RNAi in L. cuprina but we think that is because Lucilia completes cellularization quickly (about 2-3h compared to 10h in medfly), which limits how far dsRNA can diffuse from the injected end before cells form. We do produce a few viable fully transformed XX males from these transient RNAi experiments. We added a couple of sentences to the beginning of the second paragraph of the Discussion on this point and cited the landmark paper on Cctra.*

There is also a significant applied aspect to this work, as the authors note. “Sexing strains” are desirable for various genetic pest management methods, particularly sterile-male methods. The authors have previously developed such strains, and it is not obvious that the route described here would be preferable. As the authors note, a full sex-reversal system, producing fertile XX pseudo-males, could be very useful indeed, though those males would have to be sexually competitive as well as fertile (in fact for some applications sexually competitive may be more important that fertile).
In summary, a solid piece of work, no revolutionary new findings but empirical confirmation of some expectations in a demanding system of economic importance.

*We appreciate the positive comments and the recognition that doing this kind of research in non-model insects can be a challenge.*

Minor comments
Line 60 “the master gene” probably there is no “master gene”. Tra is important, dsx is important, the various things upstream of tra are certainly important and perhaps more commonly called “the master gene”. A “key” or “central” gene, perhaps.

*Agreed. Changed to central gene as tra is downstream of M but upstream of dsx and fru*

Line 96 I think “fertile and adequately sexually competitive…” rather than just fertile, though strictly perhaps longevity and other issues would be relevant also. This point comes up again in Discussion line 267.

*Agreed. We have modified the text accordingly. So far we have not seen major changes in longevity in transgenic strains but male sexual competitiveness is an issue with some strains.*

Line 137 I expected Figure 2C, cited here, to show dissections – but it does not. I’m not sure that this citation of the figure supports the statement in this sentence.

*Agreed. For the results shown in Fig 2 we only scored flies as males, female or intersex based on external morphology. The sentence has been deleted. We also don’t have any images to support the statement. We can’t easily take images now as the DR3#6; traIR12X strain was shutdown during the pandemic when access to the lab was restricted.*

Line 158 looking at the graph, expression in DR3#6… XX seems to be up relative to wild type or transgenic XY, but expression in transgenic XY appears to be slightly lower than in wild type XY. Possibly this is not significant, or perhaps there are strain differences, but perhaps worth commenting on.

*We did state that “A significant difference in expression was observed in autosomal genes comparing the same sex across strains, with higher overall expression observed in wild type (P <2.2e-16 (XY) and P = 0.002 (XX), Mann Whitney U Test) (Figure 5B)” in results and elaborated in the statistical methods section of methods. These statistical values have now been marked on Figure 5B. We are not sure why we see this difference but suggest it may be a consequence of breeding a strain that is homozygous for two transgenes. We have added a sentence to the results section.*

Plasmids: though the construction is detailed in M&M, I would have liked to see accession numbers for the key constructs/sequences. While it should be possible to unambiguously reconstruct the sequence from the M&M data, my experience is that this is generally not be possible. To be fair, I have not tried in this particular case, so this is a general observation rather than a specific criticism

*The plasmid sequences have been deposited in GenBank. The accession numbers are now included in the data availability statement.*

Reviewer #2: In this manuscript Williamson and colleagues studied the effect of RNAi-mediated knockdown and CRISPR/cas9-mediated knockout of transformer in the sheep blowfly. While knockdown was achieved by tet-dependent conditional expression of inverted repeats targeting transformer, knockout was achieved by knocking in a marker gene. Female to male sex conversion was observed in both knockdown and knockout experiments. Female-specific lethality was observed only in RNAi-mediated knockdown in some cases. The authors further showed that the female-specific lethality likely resulted from increased X-chromosome gene expression, linking transformer to dosage compensation. They also demonstrated the ability to produce all male progeny through female-to-male conversion or female lethality. Overall, the manuscript is well written and the work will attract the readership PLoS Genetics who are interested in either the fundamental biology of sex determination and dosage compensation or practical SIT applications. I only have a few minor points that should be addressed.

1) The authors did a good job discussing the importance of maternally deposited transformer. In this context, it will be beneficial to describe the promoters used in DR2-DR7. I may have missed it, is it true that XX lethality was only observed in one of DR3 lines, but not any line driven by the zygotic-only promoters? If so, the authors may want to clearly state that as it is consistent with their explanation as to why KI did not lead to X activation or XX lethality. Is it possible to compare the expression level of nbl in the wildtype XX, KI XX and DR3 XX embryos?

*We have modified figure 1 to include a summary of the different tTA drivers and their activity in embryos and ovaries. We do note that lethality was only seen with the DR3#6;traIR7 strain when homozygous for both transgenes. We also note that the DR3 differs from the other drivers in having expression in both ovaries and embryos.*

*How transformer regulates dosage compensation is clearly a major question going forward. As the referee suggests, one possibility is that tra regulates nbl as nbl is required for normal X chromosome gene expression. We have not found any evidence of sex-specific RNA splicing of nbl transcripts (Davis et al, 2018). We examined the RNAseq data from this study and found little difference in nbl RNA levels between the sexes (TPM of* 2.36554 for females and 2.39942 for males). *Given that males don’t produce TRA protein it does not appear likely that TRA directly regulates nbl expression. Nevertheless, TRA could be indirectly modulating NBL activity for example via an RNA since NBL is an RNA binding protein. We hope to continue on this project to uncover how TRA controls X chromosome gene expression in Lucilia cuprina.*

2) Figure 4: I am not sure why Y-specific primers were not used to genotype these individuals. While ddPCR works well, quantifying gene copy numbers by regular qPCR is tricky.

*Although we have invested considerable time and resources into assembly a genome for the LA07 strain of Lucilia cuprina we had until recently not succeeded in identifying any Y-linked scaffolds. We now have primer pairs that can be used to distinguish XY and XX by PCR. At the time the qPCR analysis was done we had not yet identified these primer sets. We are however confident in the qPCR analysis as we have run controls with ZsGreen or DsRed transgenes that we know are X-linked or autosomal as well L. cuprina genes that are known to be autosomal (Linger et al 2015).*

3) Figure 5: why is the X:A ratio in the wildtype females closer to 2 instead of 1? This does not impact the conclusion as the authors are comparing wt with knockdown lines. However, it is good to discuss.

*With a very small number of genes in the denominator (54) and a large number in the numerator (9271), the X:A ratio calculation is sensitive to small differences of RNA levels of X-linked genes between the sexes. The 54 X-linked genes show slightly higher RNA levels in females than males (average female: male ratio of 1.12). At this stage of development a few highly expressed X-linked genes show slightly higher RNA levels in females than males (eg CVS40_11147 TPM of 1369 in females and 1176 in males). As a consequence the X:A ratio is slightly higher in wild type females than males. A sentence to this effect has been added to the results when discussing panel 5C.*

4) I also wonder why double homozygous are needed to observe effects in the knockdown experiments. Am I mistaken?

*We do observe effects in the offspring of crosses between driver and effector lines that carry one copy of each transgene (Fig 1). Most crosses produced some XX intersexes and some did not produce any females. If the referee means female lethality, yes that was only observed in the double homozygous DR3#6; traIR7 strain. Our results suggest that with moderate knockdown of Lctra, XX develop as intersexes and with strong knockdown there is complete transformation to XX males. Only in the double homozygous DR3#6; traIR7 strain with maternal and zygotic knockdown do we see female lethality.*

5) Please also discuss the 2019 publication showing that a Y-linked Guy1 gene regulates dosage compensation in Anopheles stephensi by increasing the transcription of X-linked genes (Qi et al., eLife: <https://elifesciences.org/articles/43570>)

*We agree with the referee that this is an important finding that should be included in the discussion of genes that control both sex determination and dosage compensation. We have revised the first paragraph of the discussion to more fully discuss sex determination and dosage compensation in Anopheles mosquitoes, including the importance of Guy1.*

6) Line 264, Change “though” to “through”.

*corrected*

Reviewer #3: The study is well thought out and each experiment builds up towards showing that the transformer gene plays a major role in sex determination and dosage compensation in Lucilia cuprina.

Major concerns:
This study has attempted to create XX transformed males for an Australian sheep pest Lucilia cuprina. The central idea behind creating these transformed males was to introduce the parent transgenic flies in the wild as a genetic control program. The authors were successful in creating these flies but could not produce fertile transformed males implying that if a sterile insect technique to control L. cuprina population exists, these transgenic flies would create no different economic burden if released in the wild. Although their objective was not achieved, they have discovered the role of the transformer gene in L. cuprina. Further tweaking of the transgenic flies is needed to achieve the objective the authors set out for.

Specific concerns:
1. Line 93 – How does the presence of the first intron from the Cochliomyia hominivorax kill females?

*Thanks for drawing attention to this section. The description of the female killing system was too brief. Additional sentences have been added to more clearly describe how the system works.*

2. Line 115, Figure 1A- Schematic illustration of the traIR effector construct with the Lctra inverted repeat shows a hsp83 promoter whereas the results sections says it is under hsp70 promoter.

*The Lchsp83 promoter drives the expression of the RFPex marker gene as shown. The Lchsp70 core promoter was used in the Lctra RNAi construct but was not previously shown in the Figure. It has been added to Figure 1 prior to the inverted repeat.*

3. Numbering system of the transgenic offspring is confusing. For eg. What does “#6” and “7” in DR3#6 LctraIR7 stand for?

*This is the system we use in the lab for keeping track of transgenic lines. Line #6 would have come from G0 cross #6 from embryos injected with the DR3 plasmid. This is the numbering system we have used in all previous publications so we cannot change it for this paper. A sentence has been added to the legend of Fig 1 to explain what the numbering means.*

4. A supplementary figure with a schematic of the cross between the DR lines and IR construct would help understand the results and the numbering system better.

*A supplementary figure has been added with the crossing scheme.*

5. As the four driver lines are extensively used in the paper, a brief statement on what different promoters these individual lines carry would help distinguish the driver lines.

*The driver constructs have been added to Figure 1, noting activity in embryos or embryos + ovaries. We did explain in the first paragraph of the Results that three of the drivers use a promoter from a cellularization gene*

6. Line 124 – The authors need to mention the range of intersex phenotypes they were looking for in order to clearly understand what is considered as intersex.

*Intersex flies were scored based on the genitalia and interocular distance. The external phenotypes observed ranged from a bent ovipositor (90° bend) to full male genitalia with female interocular distance. Description of what is considered intersex has been added to the methods section.*

7. Figure4/5/6- No significance values are marked on the figures.

*Figure 4: Each unknown sample was tested for a significance difference from both the male and female control samples for both genes by Tukey’s HSD. Updated figure legend to reflect the analyses done.*

*Figure 5: Significance values added to all graphs in Figure 5. Sentence added to Results section of manuscript referencing significance shown in Figure 5D and updated figure legend.*

*Figure 6: Significance marked on 6E and sentence added to results section of manuscript referencing the significance shown in this figure. Figure legend updated.*

8. Figure3B, 6D – PCR products for doublesex M are of quite varied lengths in both the figures. A graphical representation of the band intensities in comparison to the control 28S band would clearly represent these datasets.

*With RT-PCR, we are looking for the presence of the male or female band, using a male or female specific primer set that amplifies within the doublesex gene. We are not using RT-PCR to quantify the amount of product present. The SmartGlow Loading Dye used to visualize the products produces slightly varied product sizes if there are any contaminants in the PCR product, which accounts for the slight range in sizes observed. However, product was only amplified for both the DR3#6 LctraIR7 XX pupae and LctraKI XX males using the male specific dsx primer set. There is no more RNA or cDNA left from this testing to redo the PCR and repeating the crossing scheme would take months to complete.*

9. Line 195 – why were hemi-sected adults used and not the whole body for qRT-PCR?

*The corresponding author was trained by John Lucchesi. The standard practice in the Lucchesi lab was to use hemisected adults for X chromosome dosage compensation studies as abdomens contain most of the sexually dimorphic tissue. The text has been modified to explain why we used hemisected adults with citations to earlier studies on dosage compensation from the Lucchesi and Scott labs.*

10. Figure 5D, 6E - The X axis gene order on both the graphs is different – not making it easy to compare the two figures.

*Adjusted graphs to have the same X axis gene order.*

11. Line 200 - Why were the 5 genes chosen? I am not convinced that a significant increase in 2 of the chosen 5 genes translates to an hyperactivation of the X chromosome transcription in the XX DR3#6; LctraIR7 flies.

*Line 200 is in the paragraph on the Lctra knockin line. In an earlier section we reported on the X chromosome hyperactivation observed in the XX DR3#6; LctraIR7 pupae. For DR3#6; LctraIR7 we did both RNAseq and qRT-PCR on 5 selected X-linked genes. All 5 genes showed a significant increase in expression in XX pupae (Fig 5D). We measured the RNA levels for the same 5 genes in XX adults with the Lctra knockin. Only 2 genes showed a significant increase. So, we agree that there does not appear to be widespread X chromosome hyperactivation in the Lctra knockin strain.*

Minor concerns:
This study is based on L. cuprina being a sheep pest but nowhere have the authors mentioned why it is regarded as a pest or what the economic consequences of having it as a pest are.

*The importance of L. cuprina as a pest was introduced in the fourth paragraph of the introduction. As what we wrote was perhaps too brief, additional sentences with appropriate citations have been added to give more of an introduction of why L. cuprina is such a serious pest that is widely despised by sheep farmers.*

**Have all data underlying the figures and results presented in the manuscript been provided?**
Large-scale datasets should be made available via a public repository as described in the *PLOS Genetics* [data availability policy](http://journals.plos.org/plosgenetics/s/data-availability), and numerical data that underlies graphs or summary statistics should be provided in spreadsheet form as supporting information.

Reviewer #1: Yes

Reviewer #2: Yes

Reviewer #3: Yes

PLOS authors have the option to publish the peer review history of their article ([what does this mean?](https://journals.plos.org/plosgenetics/s/editorial-and-peer-review-process#loc-peer-review-history)). If published, this will include your full peer review and any attached files.


If you choose “no”, your identity will remain anonymous but your review may still be made public.


**Do you want your identity to be public for this peer review?** For information about this choice, including consent withdrawal, please see our [Privacy Policy](https://www.plos.org/privacy-policy).

Reviewer #1: No

Reviewer #2: No

Reviewer #3: No
